# Calculating maternal polygenic risk scores from prenatal screening by cell-free DNA data

**DOI:** 10.3389/fgene.2025.1495604

**Published:** 2025-02-20

**Authors:** Victoria Corey, Mauro Chavez, Layla Qasim, Tevfik U. Dincer, Angela Henry, Salome Bagayan, Sasha Treadup, Mike Mehan, Eileen de Feo, Sung Kim

**Affiliations:** Illumina, Inc., San Diego, CA, United States

**Keywords:** polygenic risk score, prenatal screening, cell-free DNA, whole genome sequencing, imputation

## Abstract

Polygenic Risk Scores (PRS) have enabled quantification of genetic risk for many common and complex traits. Here we developed a novel method to estimate maternal PRS using low-coverage whole genome sequencing data from prenatal screening by cell-free DNA data intended to screen for fetal chromosomal aneuploidies. A prospective study was conducted where 455 consented patients that performed prenatal screening by cell-free DNA as part of their standard of care were randomly selected. Cell-free DNA and genomic DNA were isolated from the plasma and buffy coat of the blood drawn from pregnant women, respectively. Cell-free DNA was sequenced at ∼0.25x coverage while genomic DNA was sequenced at ∼15x coverage. The sequence data was used to impute genotypes which were then used to calculate PRS for paired comparisons. There was a high correlation (average = ∼0.9 across different PRS panels and panel sizes) between PRS from prenatal screening by cfDNA data and PRS from genome sequence data of the buffy coat. This proof-of-concept study illustrates that maternal PRS can be calculated using low-coverage prenatal screening by cfDNA sequence data with high accuracy.

## 1 Introduction

Polygenic risk scores (PRS) are numerically weighted summarizations of genotypic alleles of an individual that capture their genetic traits or disease risk (Choi et al., 2020). Large databases, like the United Kingdom Biobank, have enabled the dissection, evaluation and validation of PRS in concert with genome-wide association studies across many complex traits and diseases (Thompson et al., 2022). These studies move the bar to precision medicine where application of PRS can improve diagnosis of disease risk, inform clinicians in choosing therapeutics, and inspire risk-reducing lifestyle habits (Polygenic Risk Score Task Force of the International Common Disease Alliance, 2021).

Despite advances in sequencing technologies, high coverage sequencing initiatives for population scale PRS related studies remain costly. Therefore, genotyping arrays and low depth sequence data (4X coverage) are still used to conduct PRS studies to circumvent the need for cost prohibitive high coverage whole genome sequence data for all samples. To overcome challenges of missing genetic information from both approaches, statistical methods were developed to impute (probabilistic inference based on observed data) the missing genotypes (Rubinacci et al., 2023). Further, reference panels like 1,000 Genomes (The 1000 Genomes Project Consortium, 2015) Project and the multi-ethnic NHLBI Trans-Omics for Precision Medicine (TOPMed) (Taliun et al., 2021) program gave way to improve accuracy of imputation. These two advances have thus paved the way for alternative and cost-effective methods to conduct PRS studies at population scale.

One alternative and cost-effective approach may be whole genome sequencing (WGS) based Non-Invasive Prenatal Testing (NIPT). WGS NIPT sequences cell free DNA (cfDNA) isolated from the plasma of pregnant women as early as 10 weeks gestation to screen for the presence or absence of fetal chromosomal abnormalities, such as Trisomy 21 (Palomaki et al., 2011; Pertile et al., 2021). Further, WGS NIPT typically generates mostly maternal sequence reads, on average 90% (Kinnings et al., 2015), at shallow depth (∼0.25x) which may be sufficient to calculate maternal PRS. As NIPT is standard of care for hundreds of thousands of pregnancies, this may provide an interesting opportunity for population scale clinical studies to evaluate both maternal genetic risk for disease and pregnancy related condition. For example, increased hypertension risk has been linked to increased risk for pre-eclampsia and early determination of elevated genetic risk could inform and improve pregnancy management and delivery interventions (Roberts and Gammill, 2005).

In this study, we conduct a proof-of-concept study to evaluate whether WGS NIPT can be a data source for future PRS studies and methods. Specifically, we evaluate 1) whether WGS NIPT data at very low coverage (0.25x) is sufficient for accurate genotypic imputation and 2) whether maternal PRS can be calculated when cfDNA is a mixture of fetal and maternal DNA.

## 2 Materials and methods

### 2.1 In-silico study

The accuracy of maternal PRS calculations using whole genome sequence data from prenatal screening by cfDNA results have two challenges: 1) low sequence coverage of ∼0.25x and 2) mixture of fetal and maternal genetic information.

To assess the impact of low sequence coverage, 10 samples from the 1000 Genomes Project (The 1000 Genomes Project Consortium, 2015) were *in silico* down sampled using FASTQ files to generate sequence coverages of 0.2x and 1.0x (Supplementary Table S1). For down sampled data with less than 30x coverage, the following steps outline bioinformatic analysis pipeline. First, use DRAGEN™ v4.0 Germline with forced genotyping to generate vcf files for low coverage data. Second, apply DRAGEN Imputation v4.2.4 to generate imputed vcf files. The vcf files from imputed 0.2x, imputed 1.0x, and 30X samples were then processed by PLINK (Purcell et al., 2007) (v. 1.07) to calculate PRS. In brief, PRS is the sum of calculated weights conditional on the presence or absence of a specific genotype marker. Specifically, $\mathrm{PR}S_{j}=\frac{\sum_{i}^{N}\beta_{i}*G_{ij}}{M_{j}}$ where for each sample *j*, and each SNP *i = 1, … ,N*, the sum of each weighted ( β ) imputed genotype ( G ) are normalized by a scalar parameter ( M ). 2

Forced genotyping is a necessary step that enables genotyping calls at pre-specified genomic positions pre-identified by the DRAGEN Imputation reference panel v1. This is in part critical due to the extremely low coverage data. Accuracy of PRS were then assessed by comparing scores generated from 0.2x and 1.0x data relative to 30x data.

To assess the impact of using maternal sequence data confounded by fetal genetic information, paired mother and child from the 1000 Genomes Project (The 1000 Genomes Project Consortium, 2015) were *in silico* mixed to emulate prenatal screening by cfDNA data. Sequence reads from 2 paired mother and child (NA12877 and NA12878) were mixed at ratios of 100:0, 95:5, 90:10 and 85:15 respectively to mimic a range of fetal fractions observed in plasma of pregnant women (Kinnings et al., 2015). The *in silico* mixtures were then down sampled to create 7 replicates for each mixture with 0.25X coverage and processed through the bioinformatic pipeline described above. This allowed for comparisons of PRS scores from the maternal genotypes with and without average fetal genotypic contributions expected in samples derived from prenatal screening by cfDNA testing.

### 2.2 Prospective study

A prospective study was conducted where 450 consented de-identified patients that performed prenatal screening by cfDNA as part of their standard of care for their pregnancy were randomly selected. The study was intentionally designed with samples that screened negative for fetal aneuploidy to assess practicality of maternal PRS calculation. The remnant patient samples were initially collected by Illumina Laboratory Services, Foster City, CA during the period of September 2022 to December 2022 for the NIPT laboratory developed test (LDT). Supplementary Table S2 describes basic demographic information obtained during patient intake. To generate the NIPT data, cfDNA was isolated from the plasma of the blood drawn from pregnant women and was processed by an LDT adaptation of the VeriSeq™ NIPT Solution v2 (Pertile et al., 2021) The cfDNA was sequenced at 48-plexity to generate ∼0.25x genome-wide coverage per sample on NextSeq™ 550.

gDNA was subsequently isolated from the buffy coat from the same blood samples using a QIAsympony SP instrument and QIAsymphony DSP DNA Midi Kit. The extracted gDNA was fragmented using a Covaris LE220-Plus sonicator and converted into libraries using automated methods of the TruSeq™ DNA Nano High Throughput Library Preparation kit. The libraries were quantified using qPCR KAPA library quantification kits and normalized to 1.5 nM. To generate the maternal genetic data, the normalized libraries were combined into 64-plexity pools and sequenced on NovaSeq™ 6,000 using S4 flowcells and using the NovaSeq 6,000 xP workflow, resulting in ∼15x genome-wide coverage per sample. Identical to the *in silico* study described above, all sequence data were analysed using DRAGEN™ v4.0 and imputed by DRAGEN Imputation v4.2.4. PRS was then calculated using PLINK which utilized the imputed genotypes and 100 PRS panels from the Polygenic Score Catalogue (https://www.pgscatalog.org). Selected pregnancy-related phenotypes for PRS calculations include diabetes, hypertension, and breast cancer (see Supplementary Table S3 for list of PRS panels evaluated).

## 3 Results

### 3.1 *In-silico* study results

**FIGURE 1:**
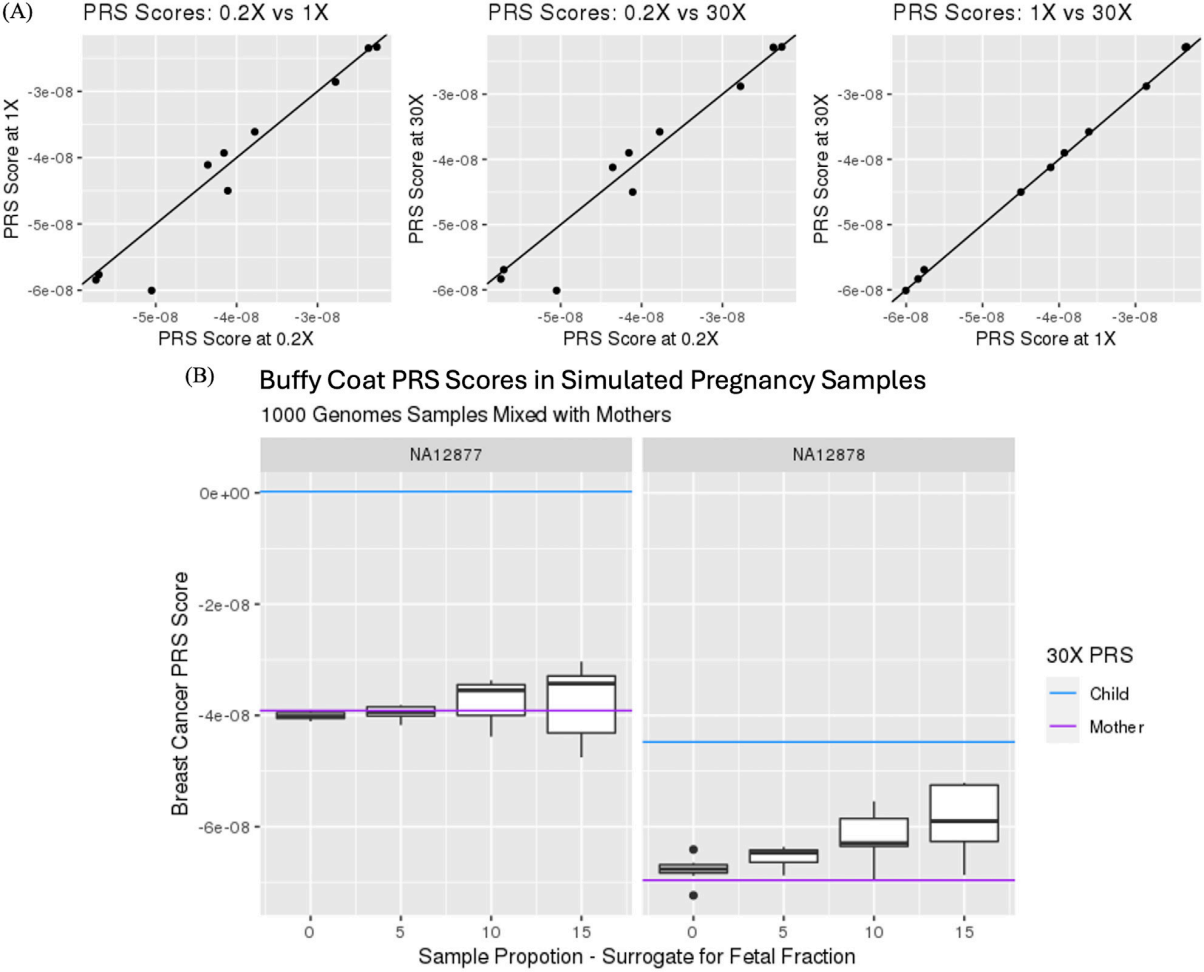
**(A)** Comparison of polygenic risk scores (PRS) for PGS000332 from imputed genotypes using 0.2X and 1.0X sequence coverage data and non-imputed 30X sequence coverage data. Correlations were 2:0.999 for lX vs 30X (left), 0.968 for 0.2X vs 30X (middle) and 0.970 for 0.2X vs LOX (right). **(B)** Impact of maternal PRS with and without in silico mixture of 0, 5, 10 and 15% fetal and maternal genotype information.

Ten samples from the 1,000 Genomes study were selected for *in silico* down sampling to assess the impact of calculating PRS from sequence coverages that mimic prenatal screening by cfDNA testing. Figure 1A illustrates the 3-way comparison of PRS for breast cancer (PGS000332) calculated using 0.2X, 1.0X and 30X sequence coverage data. Correlation of PRS was highest when comparing imputed 1X to non-imputed 30X genotype data (corr ≥0.999) and lowest when comparing imputed 0.2X to imputed 1X genotype data (corr ≥0.970). This slight reduction in correlation is likely a result of reduction in genotype imputation accuracy due to the reduction of sequence coverage data, i.e., genetic information content, from 30X to 0.2X.

Calculation of maternal PRS using prenatal screening by cfDNA data is not straight forward as the sequence contains both maternal and fetal genotype information. By generating *in silico* mixtures, the impact of the fetal genotype on maternal PRS can be assessed at different levels of fetal fraction. Figure 1B highlights the impact of adding fetal genotype information on maternal PRS score calculations. Specifically, the impact of fetal genotype increases variability and bias of PRS as fetal contribution increases in its calculation. Nonetheless, correlation between mixtures remained high when comparing 0%–5%, 0%–10% and 0%–15% with correlation measures of 0.985, 0.942 and 0.875, respectively. These *in silico* studies support and provide simulated evidence that PRS from whole genome sequencing in the context of a prenatal screening by cfDNA testing is feasible.

### 3.2 Prospective study results

**FIGURE 2:**
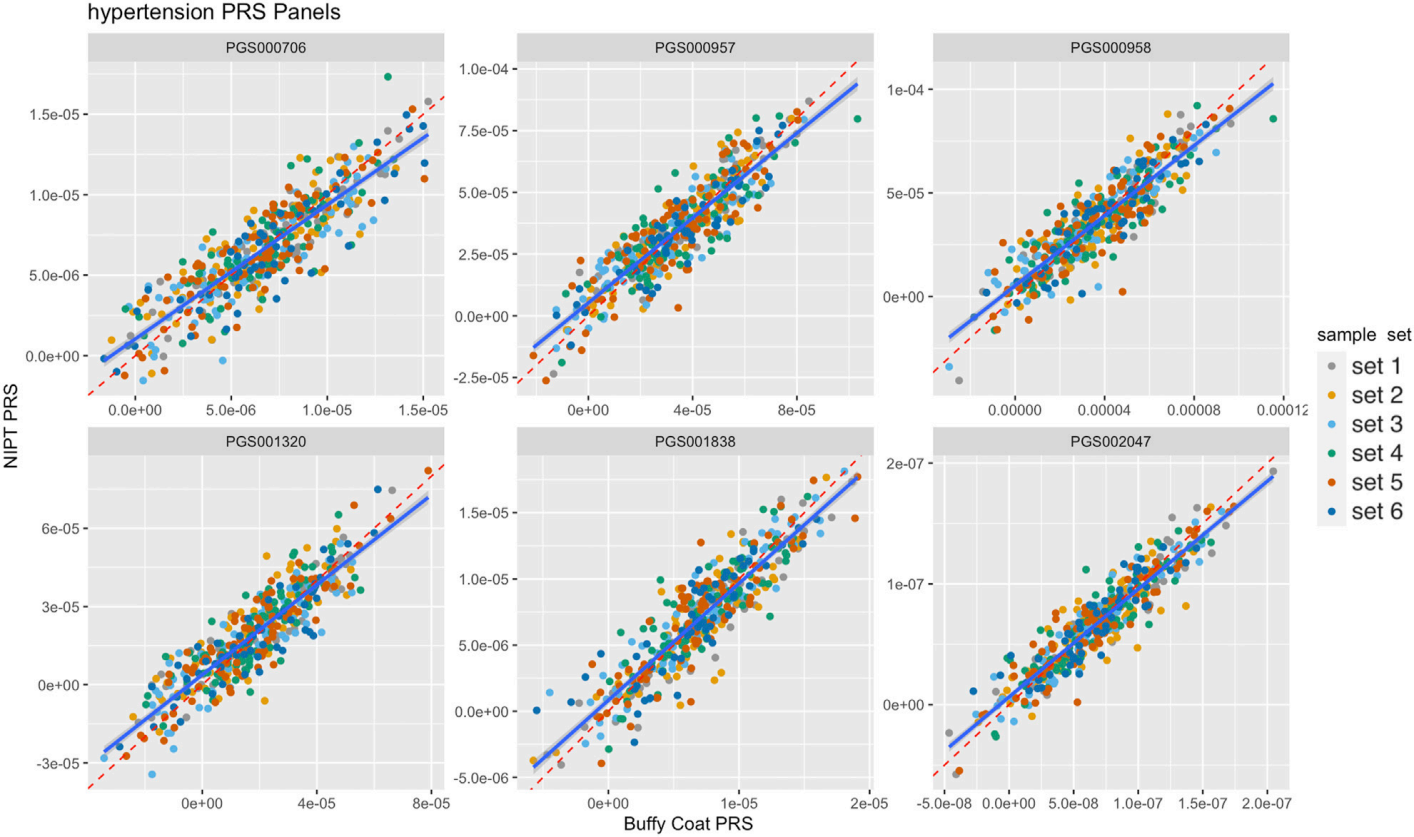
Comparison of six different hypertension polygenic risk score (PRS) panels from buffy coat genomic DNA (gDNA) and NIPT cell-free DNA (cfDNA). Red­dashed and blue lines denote the identity line and the fitted linear model, respectively. Colors denote flow cell batches, i,e, sample sets.

**FIGURE 3:**
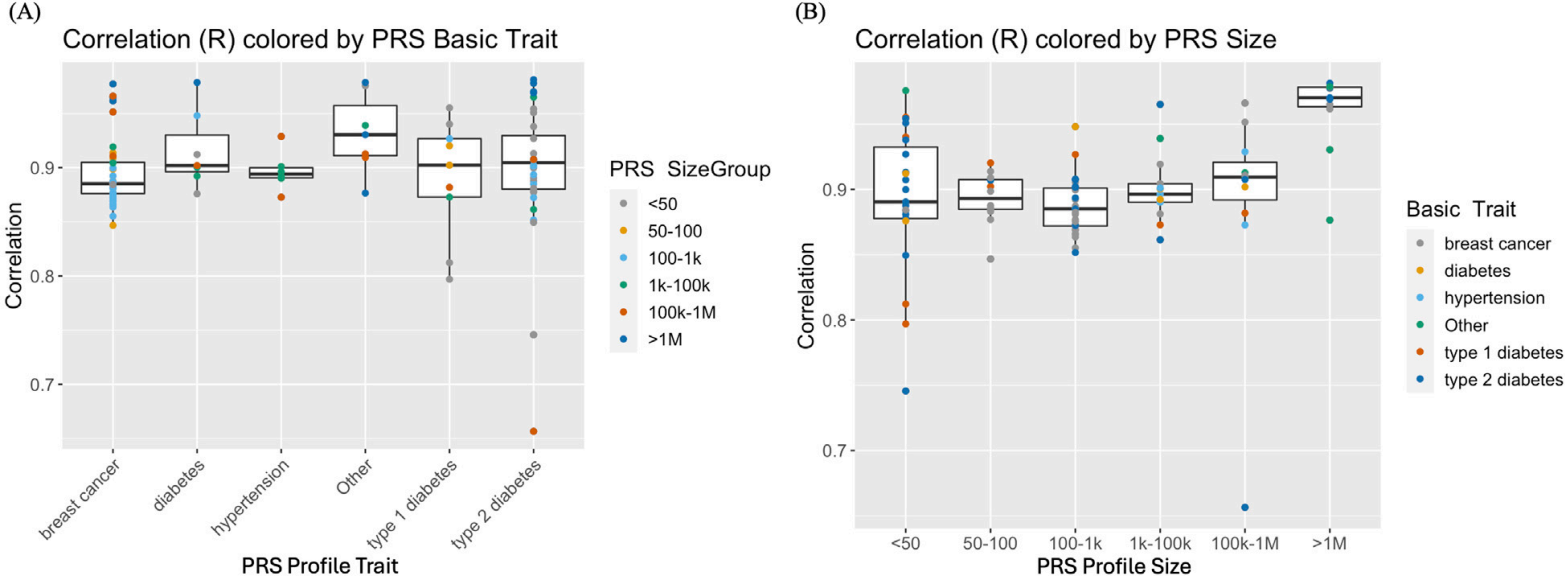
**(A)** Correlation of polygenic risk scores (PRS) from genome sequence data of the buffy coat vs PRS from prenatal screening by cell-free DNA (cfDNA) data. PRS panels of breast cancer, type I or type 11 diabetes, hypertension and “Other” (that include arthritis and coronary artery disease) with varying PRS panel sizes ranging from <50 to >IM genetic markers. **(B)** Comparison of PRS correlations conditional on PRS panel/profile size.

Imputed genotype data generated from analyzing whole genome sequence data of cfDNA and gDNA isolated from the blood of pregnant women were used to demonstrate proof of concept evidence in the feasibility of calculating maternal PRS. Figure 2 illustrates paired scores from 6 different PRS hypertension panels calculated using sequence data from gDNA and cfDNA. The correlation for the 6 PRS ranged from 0.87 to 0.93 consistent with the observed simulated results. Overall, there was high correlation (average = ∼0.90 across different PRS panels and panel sizes) between PRS from prenatal screening by cfDNA data and PRS from genome sequence data of the buffy coat (Figure 3A; Supplementary Table S3). Furthermore, panel size had on average a minor effect where panels with <1M markers had correlation ∼0.90 whereas panels with >1M markers had correlation ∼0.95 (Figure 3B).

**FIGURE 4:**
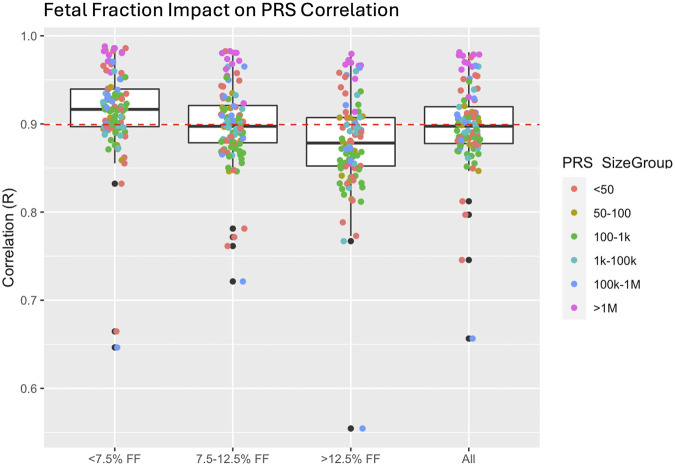
Impact of fetal fraction (FF) on polygenic risk score (PRS) correlation. Higher FF resulted in a slight reduction in correlation in PRS most likely due to reduction in imputation accuracy resulting from the presence of the fetal haplotype.

Although PRS comparisons demonstrated high concordance, estimating maternal PRS from prenatal screening by cfDNA data is suboptimal because the sequence data contains genetic information from both mother and fetus. Specifically, genetic information from the fetus may confound the accuracy of PRS as the presence of fetal alleles may lead to imputation errors. This is illustrated by higher PRS correlations (mean ∼ = 0.92) in samples with lower fetal fraction (FF) compared to samples with higher FF (mean correlation ∼ = 0.88) (Figure 4). We estimated the impact of this confounder on risk categorization for clinical applications to be as low as 2% (Supplementary Figure S1) for previously proposed high-risk cutoffs of 5% of the population (Lennon et al., 2024).

## 4 Discussion

Understanding and providing early detection of genetic risk is intended to improve patient care and health management. Indeed, availability of large-scale cohort databases with aims of enabling PRS to quantify and predict genetic risk for many common and rare/complex traits has increased. However, biobanks and large-scale databases often rely on population scale initiatives for genomics which can lead to ascertainment biases, limited opportunities in adoption for participants, and unknown and inaccurate clinical predictive power of PRS panels especially in under-represented populations. However, as more studies directly tackle these issues (Chen et al., 2020; Martin et al., 2021; Duncan et al., 2019), PRS accuracy, diversity, and application will improve over time as more data becomes available.

One alternative to this paradigm is leveraging pre-existing high throughput standard of care workflows built around next-generation sequencing technologies. Low-coverage prenatal screening by cfDNA sequencing is one such avenue. In many countries, 25%–50% of women have performed prenatal screening by cfDNA (Gadsbøll et al., 2020). This scale and utilization provide direct opportunities to 1) create new large-scale database for PRS improvements without additional costs to healthcare systems or funding for large-scale genomics 2) enables retrospective and prospective studies by evaluating PRS against electronic medical records to establish analytical validity and clinical utility 3) provide direct and early intervention and adoption for improved pregnancy management.

The goal for this proof-of-concept study was to illustrate that maternal PRS can be calculated using low-coverage prenatal screening by cfDNA sequence data intended to screen for fetal chromosomal aneuploidies with high accuracy. This study was not designed to consider or evaluate the robustness of imputation or PRS in a population but rather the reproducibility of a PRS score at an individual level from a novel data source. Ultimately, maternal PRS may be used to improve patient and pregnancy care as genetic data from prenatal screening by cfDNA can be applied to many other PRS panels that are designed to find genetic risk factors for specific phenotypes. For example, there is evidence that genetic risk for hypertension or type II diabetes have been implicated to increase patient’s risk of pre-eclampsia and gestational diabetes, respectively. Identifying patients as early as 10 weeks gestation that are potentially high risk for either of these conditions could be beneficial.

Although the aims of the study were specifically to assess the technical feasibility of calculating maternal PRS from sequence data generated by prenatal screening by cfDNA testing, this study outlined two limitations. First is the impact of FF and the bias of maternal PRS. As our results suggest, FF is a confounding factor in the accuracy of maternal PRS. However, the overall magnitude of the effect is likely small for clinical applications of PRS as PRS typically relies on validated risk stratification thresholds. Predicted misclassification of NIPT-based PRS scores relative to maternal PRS scores are on the order of 2%–8%. A potential bioinformatic solution that leverages known fragment length differences between maternal and fetal cfDNA may be applied; fetal cfDNA is on average shorter than maternal cfDNA. Lo et al. found maternal cfDNA to have a peak at 166-base pairs (bp) while fetal cfDNA has a peak at 143-bp (Lo et al., 2010), and this physical size difference may provide an opportunity to improve maternal PRS calculation. However, the *in silico* removal of shorter fragment length sequences prior to imputation with the intent to reduce fetal signal did not reduce the bias of imputation incurred by the presence of the fetal alleles. This suggests that more sophisticated methodologies may be warranted or that the reduction of sequence and genetic information from down-sampling NIPT data outpaces the expected reduction of fetal bias in PRS calculation.

Second, clinical validation and follow up is limited as the study was conducted with consented de-identified screen negative patient samples. Though clinical results are not necessary for this technical feasibility study, the clinical indications for PRS would benefit greatly from its utility for patient and pregnancy health management. In addition, evaluating samples with positive fetal aneuploidy could also help better understand overall robustness in maternal PRS calculations. Further work on obtaining clinical follow up and test indications is warranted to demonstrate how PRS panels can predict genetic risk factors for pregnancy related conditions such as pre-eclampsia and gestational diabetes.

## Data Availability

The raw data supporting the conclusions of this article will be made available by the authors, without undue reservation.
