# Supplementary material for: Calculating maternal polygenic risk scores from prenatal screening by cell-free DNA data
Source: Front Genet. 2025 Feb 20;16:1495604. doi: 10.3389/fgene.2025.1495604 (PMC11882851; doi:10.3389/fgene.2025.1495604)

Supplementary Material

# Supplementary Figures and Tables

Supplementary Table 1 – List of 1000 Genomes samples used for *in silico* study.

| Study | Sample ID | 1000 Genomes Data Link |
| --- | --- | --- |
| coverage down sample | HG00185 | <https://www.internationalgenome.org/data-portal/sample/HG00185> |
| coverage down sample | HG00272 | <https://www.internationalgenome.org/data-portal/sample/HG00272> |
| coverage down sample | HG00280 | <https://www.internationalgenome.org/data-portal/sample/HG00280> |
| coverage down sample | HG00310 | <https://www.internationalgenome.org/data-portal/sample/HG00310> |
| coverage down sample | HG00315 | <https://www.internationalgenome.org/data-portal/sample/HG00315> |
| coverage down sample | HG00325 | <https://www.internationalgenome.org/data-portal/sample/HG00325> |
| coverage down sample | HG00349 | <https://www.internationalgenome.org/data-portal/sample/HG00349> |
| coverage down sample | HG00353 | <https://www.internationalgenome.org/data-portal/sample/HG00353> |
| coverage down sample | HG00368 | <https://www.internationalgenome.org/data-portal/sample/HG00368> |
| coverage down sample | HG00375 | <https://www.internationalgenome.org/data-portal/sample/HG00375> |
| mother+child mix | NA12877 (child)  NA12890 (mother) | <https://www.internationalgenome.org/data-portal/sample/NA12877>  <https://www.internationalgenome.org/data-portal/sample/NA12890> |
| mother+child mix | NA12878 (child)  NA12892 (mother) | <https://www.internationalgenome.org/data-portal/sample/NA12878>  <https://www.internationalgenome.org/data-portal/sample/NA12892> |

Supplementary Table 2: Sample Demographics

| Cohort Size | 450 |
| --- | --- |
| Maternal age, yr  Average  Median  Range | 31.90  32.00  14.33 – 45.75 |
| GA at time of sample collection, wk  Average  Median  Range | 12.80  12  10– 33 |
| BMI  Average  Median  Range | 25.59  24.36  16.41 – 53.91 |
| Fetal Fraction,  Average  Median  Range | 10.36%  9.73%  3.03% – 30.06% |

BMI, body mass index; wk, week; yr, year.

Supplementary Table 3 – Buffy coat to NIPT Correlation by PRS Panel

| PRS Panel | Trait | Panel Size | Correlation R | Low FF Correlation R | Med FF Correlation R | High FF Correlation R |
| --- | --- | --- | --- | --- | --- | --- |
| PGS000001 | breast cancer | 77 | 0.8876102 | 0.8929681 | 0.8904838 | 0.8773863 |
| PGS000002 | breast cancer | 77 | 0.8853163 | 0.890734 | 0.8907127 | 0.8693853 |
| PGS000003 | breast cancer | 77 | 0.8769327 | 0.8942145 | 0.8808352 | 0.8499931 |
| PGS000004 | breast cancer | 311 | 0.8718552 | 0.896351 | 0.8737887 | 0.8411205 |
| PGS000007 | breast cancer | 3784 | 0.881265 | 0.9218749 | 0.8827379 | 0.8274324 |
| PGS000028 | breast cancer | 86 | 0.913792 | 0.8759924 | 0.9348674 | 0.9071702 |
| PGS000045 | breast cancer | 87 | 0.885277 | 0.907401 | 0.8966733 | 0.8413125 |
| PGS000046 | breast cancer | 86 | 0.8832614 | 0.9072834 | 0.9024929 | 0.8145601 |
| PGS000047 | breast cancer | 51 | 0.8467281 | 0.858812 | 0.8463121 | 0.834533 |
| PGS000050 | breast cancer | 44 | 0.883812 | 0.9196586 | 0.8810515 | 0.8524018 |
| PGS000051 | breast cancer | 67 | 0.8985913 | 0.9084309 | 0.889752 | 0.9071392 |
| PGS000052 | breast cancer | 159 | 0.8853857 | 0.9229561 | 0.8685529 | 0.8732336 |
| PGS000072 | breast cancer | 187 | 0.9054892 | 0.9041285 | 0.9003599 | 0.9140823 |
| PGS000153 | breast cancer | 66 | 0.9089488 | 0.9161082 | 0.9187051 | 0.880023 |
| PGS000212 | breast cancer | 308 | 0.8809966 | 0.9005444 | 0.890451 | 0.8496892 |
| PGS000213 | breast cancer | 308 | 0.8923782 | 0.9200383 | 0.8902385 | 0.8642719 |
| PGS000214 | breast cancer | 308 | 0.8852633 | 0.9166301 | 0.89176 | 0.8395389 |
| PGS000215 | breast cancer | 308 | 0.8768779 | 0.9153683 | 0.8650572 | 0.8558873 |
| PGS000216 | breast cancer | 308 | 0.855229 | 0.875842 | 0.846235 | 0.8484679 |
| PGS000317 | breast cancer | 178 | 0.8689089 | 0.8711798 | 0.8775477 | 0.8588628 |
| PGS000332 | breast cancer | 6390334 | 0.9773228 | 0.9825933 | 0.9737301 | 0.9795277 |
| PGS000335 | breast cancer | 1079067 | 0.9616839 | 0.9701016 | 0.954752 | 0.966054 |
| PGS000344 | breast cancer | 267 | 0.8659815 | 0.8898167 | 0.8720836 | 0.828111 |
| PGS000346 | breast cancer | 267 | 0.8695006 | 0.9002016 | 0.8556796 | 0.8599589 |
| PGS000347 | breast cancer | 267 | 0.865757 | 0.8908942 | 0.8759554 | 0.819827 |
| PGS000508 | breast cancer | 1120405 | 0.9650474 | 0.9710575 | 0.9622194 | 0.9664053 |
| PGS000511 | breast cancer | 118374 | 0.9515126 | 0.9509305 | 0.9498847 | 0.9554624 |
| PGS000512 | breast cancer | 285846 | 0.9661232 | 0.9705202 | 0.9651947 | 0.9637741 |
| PGS000540 | breast cancer | 7116 | 0.9192696 | 0.9345929 | 0.9170483 | 0.90742 |
| PGS000773 | breast cancer | 179 | 0.8856153 | 0.9203683 | 0.8712396 | 0.8718908 |
| PGS000774 | breast cancer | 179 | 0.887289 | 0.9164061 | 0.8798306 | 0.8697326 |
| PGS000775 | breast cancer | 179 | 0.8730773 | 0.9172368 | 0.8502805 | 0.859751 |
| PGS000783 | breast cancer | 162 | 0.8996197 | 0.932457 | 0.8848279 | 0.8911894 |
| PGS000873 | breast cancer | 132 | 0.8753868 | 0.9089972 | 0.8671315 | 0.8527195 |
| PGS001336 | breast cancer | 529 | 0.8824345 | 0.9149724 | 0.8692189 | 0.8718726 |
| PGS001337 | breast cancer | 184 | 0.8636204 | 0.8982866 | 0.8631502 | 0.8260954 |
| PGS001778 | breast cancer | 15 | 0.8843462 | 0.9163388 | 0.8853845 | 0.8396535 |
| PGS001804 | breast cancer | 2984 | 0.9044057 | 0.9247946 | 0.8957701 | 0.8993159 |
| PGS002015 | breast cancer | 488608 | 0.9107488 | 0.925474 | 0.9095653 | 0.901068 |
| PGS000819 | diabetes | 3537914 | 0.9787325 | 0.9859391 | 0.9803915 | 0.9673786 |
| PGS001327 | diabetes | 3971 | 0.8922922 | 0.896136 | 0.8852574 | 0.9002772 |
| PGS001329 | diabetes | 2221 | 0.9003101 | 0.9177189 | 0.8976151 | 0.8898544 |
| PGS001371 | diabetes | 22 | 0.9122 | 0.9342788 | 0.9107987 | 0.8907084 |
| PGS001819 | diabetes | 249 | 0.9480706 | 0.9530944 | 0.9527415 | 0.9368954 |
| PGS002027 | diabetes | 389029 | 0.9019507 | 0.918647 | 0.9094528 | 0.872051 |
| PGS002256 | diabetes | 4 | 0.8759017 | 0.8971834 | 0.8949828 | 0.813195 |
| PGS000021 | type 1 diabetes | 33 | 0.9401694 | 0.9677059 | 0.9431797 | 0.9068663 |
| PGS000022 | type 1 diabetes | 37 | 0.8122542 | 0.8619336 | 0.7614531 | 0.8360194 |
| PGS000023 | type 1 diabetes | 7 | 0.9552971 | 0.9659616 | 0.9576731 | 0.9416047 |
| PGS000024 | type 1 diabetes | 85 | 0.9022961 | 0.9295388 | 0.8797191 | 0.9085746 |
| PGS000869 | type 1 diabetes | 48 | 0.7969974 | 0.8322333 | 0.7812697 | 0.7883554 |
| PGS001294 | type 1 diabetes | 3422 | 0.8728222 | 0.8971495 | 0.8642641 | 0.8550088 |
| PGS001297 | type 1 diabetes | 65 | 0.9202785 | 0.9418266 | 0.9178952 | 0.9035326 |
| PGS001817 | type 1 diabetes | 825 | 0.9267638 | 0.9339442 | 0.9281786 | 0.921922 |
| PGS002025 | type 1 diabetes | 106800 | 0.8818621 | 0.9002195 | 0.8837054 | 0.8712664 |
| PGS000020 | type 2 diabetes | 7502 | 0.9651402 | 0.9714077 | 0.9649814 | 0.9598567 |
| PGS000031 | type 2 diabetes | 62 | 0.9070423 | 0.9174668 | 0.9077442 | 0.8940441 |
| PGS000032 | type 2 diabetes | 20 | 0.8774031 | 0.8954489 | 0.8672619 | 0.8792741 |
| PGS000033 | type 2 diabetes | 10 | 0.9269502 | 0.9508204 | 0.9319736 | 0.8957721 |
| PGS000036 | type 2 diabetes | 171249 | 0.656505 | 0.6464543 | 0.7213137 | 0.5544578 |
| PGS000125 | type 2 diabetes | 40 | 0.8877252 | 0.9017356 | 0.90546 | 0.8321658 |
| PGS000330 | type 2 diabetes | 6426667 | 0.9701998 | 0.9814606 | 0.9680465 | 0.9638869 |
| PGS000729 | type 2 diabetes | 2015615 | 0.9779748 | 0.9804822 | 0.9806405 | 0.9724512 |
| PGS000804 | type 2 diabetes | 578 | 0.9075783 | 0.9285451 | 0.9149726 | 0.8682118 |
| PGS000805 | type 2 diabetes | 578 | 0.9076484 | 0.9274694 | 0.9166633 | 0.8663171 |
| PGS000806 | type 2 diabetes | 578 | 0.8850196 | 0.9010064 | 0.9050433 | 0.8325104 |
| PGS000807 | type 2 diabetes | 578 | 0.9079256 | 0.9144067 | 0.9100466 | 0.8974188 |
| PGS000808 | type 2 diabetes | 578 | 0.9022015 | 0.9288301 | 0.9060956 | 0.8679706 |
| PGS000832 | type 2 diabetes | 384 | 0.851671 | 0.8816481 | 0.8602677 | 0.811809 |
| PGS000848 | type 2 diabetes | 6 | 0.9543032 | 0.9829829 | 0.9492341 | 0.9346226 |
| PGS000849 | type 2 diabetes | 3 | 0.8495358 | 0.8552866 | 0.847695 | 0.8500265 |
| PGS000850 | type 2 diabetes | 16 | 0.9073692 | 0.928458 | 0.9093927 | 0.8855705 |
| PGS000851 | type 2 diabetes | 37 | 0.880936 | 0.8715454 | 0.8822448 | 0.8876189 |
| PGS000852 | type 2 diabetes | 8 | 0.8904625 | 0.9394152 | 0.8704352 | 0.8810005 |
| PGS000853 | type 2 diabetes | 21 | 0.7456547 | 0.6646232 | 0.7716098 | 0.7729654 |
| PGS000854 | type 2 diabetes | 27 | 0.8779311 | 0.8859229 | 0.8790048 | 0.872246 |
| PGS000855 | type 2 diabetes | 18 | 0.9379523 | 0.9577449 | 0.942446 | 0.9133655 |
| PGS000856 | type 2 diabetes | 3 | 0.9130866 | 0.9608317 | 0.9192589 | 0.8518506 |
| PGS000857 | type 2 diabetes | 4 | 0.9508613 | 0.9859858 | 0.9292626 | 0.9532451 |
| PGS000858 | type 2 diabetes | 6 | 0.8998909 | 0.903688 | 0.902639 | 0.8961505 |
| PGS000868 | type 2 diabetes | 221 | 0.8723406 | 0.895329 | 0.8802633 | 0.8330256 |
| PGS001295 | type 2 diabetes | 373 | 0.8935671 | 0.9161131 | 0.8970506 | 0.86362 |
| PGS001296 | type 2 diabetes | 340 | 0.9015142 | 0.9094284 | 0.9066744 | 0.883322 |
| PGS001357 | type 2 diabetes | 2996761 | 0.9691086 | 0.9781643 | 0.9717043 | 0.9512247 |
| PGS001781 | type 2 diabetes | 1091648 | 0.9813431 | 0.9879395 | 0.9826012 | 0.9711791 |
| PGS001818 | type 2 diabetes | 30745 | 0.8614495 | 0.8726667 | 0.8927201 | 0.7670198 |
| PGS002026 | type 2 diabetes | 830783 | 0.9076479 | 0.9292853 | 0.9201377 | 0.8585169 |
| PGS000706 | hypertension | 185068 | 0.8727284 | 0.8919981 | 0.8654746 | 0.8627071 |
| PGS000957 | hypertension | 11134 | 0.896307 | 0.8868957 | 0.9019588 | 0.8943431 |
| PGS000958 | hypertension | 9282 | 0.8919697 | 0.8879203 | 0.8945059 | 0.8918002 |
| PGS001320 | hypertension | 13602 | 0.8901948 | 0.9079882 | 0.8877272 | 0.8736776 |
| PGS001838 | hypertension | 52487 | 0.9010714 | 0.8977343 | 0.9037544 | 0.8952697 |
| PGS002047 | hypertension | 918325 | 0.9288454 | 0.9328345 | 0.9310738 | 0.9213431 |
| PGS000013 | Other | 3366036 | 0.8765176 | 0.9048157 | 0.8758328 | 0.8546084 |
| PGS000114 | Other | 26 | 0.9756792 | 0.9836021 | 0.9825476 | 0.9580673 |
| PGS000712 | Other | 182067 | 0.909433 | 0.9387161 | 0.8991578 | 0.893293 |
| PGS000713 | Other | 182202 | 0.9127615 | 0.9400819 | 0.9040237 | 0.8978302 |
| PGS999913 | Other | 3366036 | 0.9304623 | 0.9584871 | 0.9233961 | 0.9137774 |
| PGS999936 | Other | 55890 | 0.9390112 | 0.9598535 | 0.9326523 | 0.9329199 |
| PGSXXX001 | Other | 1584434 | 0.9787523 | 0.9860736 | 0.9807363 | 0.969381 |


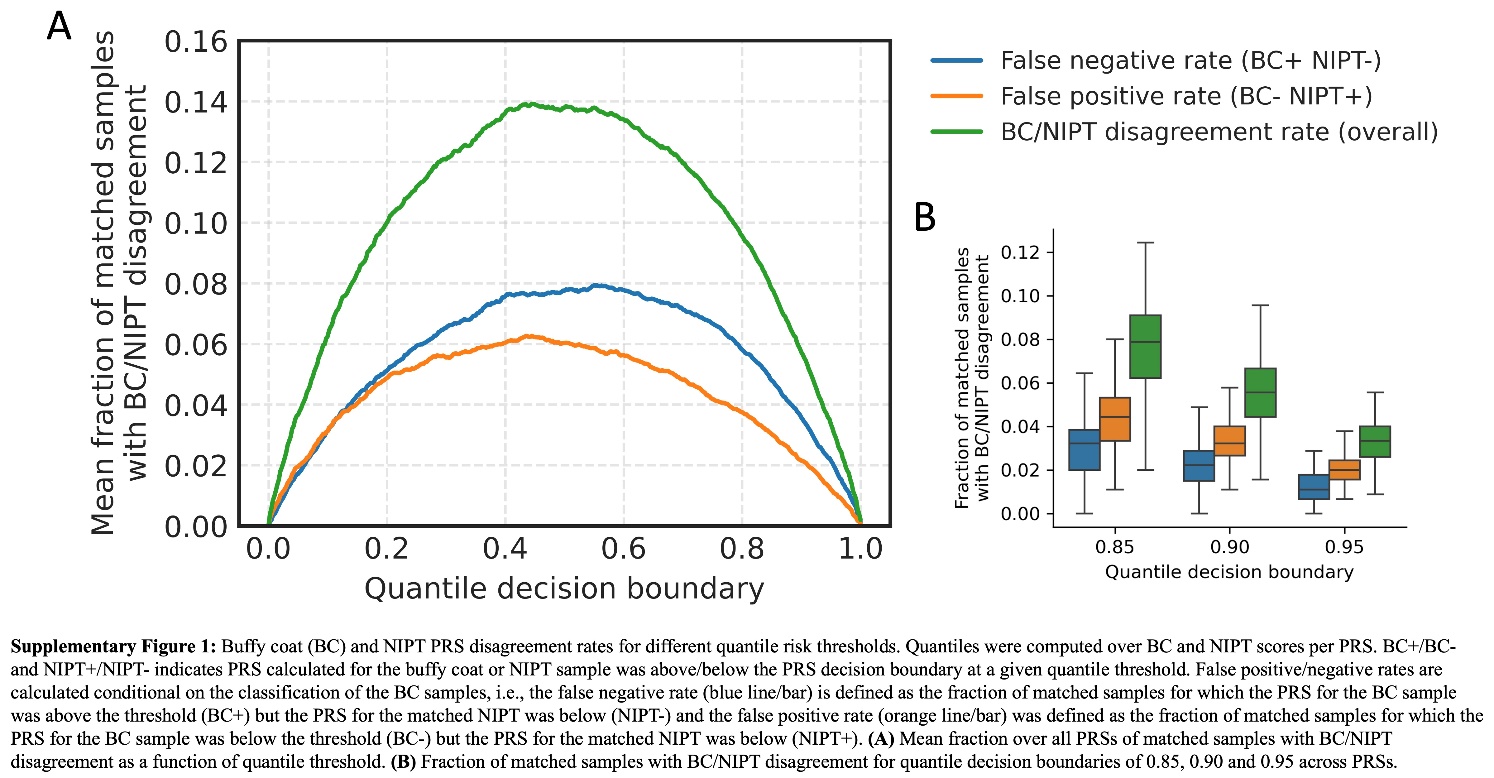

Supplement: Supplementary file 1 [file Table1.docx]
